# Phospho-kinase profile of triple negative breast cancer and androgen receptor signaling

**DOI:** 10.1186/1471-2407-14-302

**Published:** 2014-04-30

**Authors:** María D Cuenca-López, Juan C Montero, Jorge C Morales, Aleix Prat, Atanasio Pandiella, Alberto Ocana

**Affiliations:** 1 Translational Cancer Research Unit, Albacete University Hospital, 02006 Albacete, Spain; 2 Salamanca Cancer Research Center-CSIC, Salamanca, Spain; 3 Vall D’Hebron Institute of Oncology (VHIO), Barcelona, Spain

**Keywords:** Tyrosine Kinase Receptor, EGFR/PDFGRβ, PI3K-mTor, Erk1/2, TNBC (triple negative breast cancer), Kinase Inhibitors, AR (Androgen Receptor)

## Abstract

**Background:**

The androgen receptor (AR) plays a central role in the oncogenesis of different tumors, as is the case in prostate cancer. In triple negative breast cancer (TNBC) a gene expression classification has described different subgroups including a luminal androgen subtype. The AR can be controlled by several mechanisms like the activation of membrane tyrosine kinases and downstream signaling pathways. However little is known in TNBC about how the AR is modulated by these mechanisms and the potential therapeutic strategists to inhibit its expression.

**Methods:**

We used human samples to evaluate the expression of AR by western-blot and phospho-proteomic kinase arrays that recognize membrane tyrosine kinase receptors and downstream mediators. Western-blots in human cell lines were carried out to analyze the expression and activation of individual proteins. Drugs against these kinases in different conditions were used to measure the expression of the androgen receptor. PCR experiments were performed to assess changes in the AR gene after therapeutic modulation of these pathways.

**Results:**

AR is present in a subset of TNBC and its expression correlates with activated membrane receptor kinases-EGFR and PDGFRβ in human samples and cell lines. Inhibition of the PI3K/mTOR pathway in TNBC cell lines decreased notably the expression of the AR. Concomitant administration of the anti-androgen bicalutamide with the EGFR, PDGFRβ and Erk1/2 inhibitors, decreased the amount of AR compared to each agent given alone, and had an additive anti-proliferative effect. Administration of dihydrotestosterone augmented the expression of AR that was not modified by the inhibition of the PI3K/mTOR or Erk1/2 pathways. AR expression was posttranscriptionally regulated by PI3K or Erk1/2 inhibition.

**Conclusion:**

Our results describe the expression of the AR in TNBC as a druggable target and further suggest the combination of bicalutamide with inhibitors of EGFR, PDGFRβ or Erk1/2 for future development.

## Background

The understanding of the heterogeneity of cancer is of great importance for the development of effective therapeutic strategies in specific subgroups of patients. Indeed, genomic studies have classified breast cancer into different subtypes [1]. At a clinical level, this heterogeneity corresponds to different prognosis, patterns of relapse, response to treatment and clinical behavior for each breast cancer subtype. For instance, tumors expressing estrogen receptors (ER) have a more benevolent behavior compared with those overexpressing HER2 [2]. In addition, triple negative breast cancer (TNBC) shows a worse prognosis compared with tumors expressing ER, and the probability of early relapse during the first years after the diagnosed is higher compared with other subtypes [2,3].

The influence of sex hormones in the development of breast and prostate cancer is known. Estrogens and androgens act through their receptors by a direct transcriptional regulation of genes involved in cell growth and survival [4]. In breast cancer, more than 70%–80% of tumors express the ER or progesterone receptor (PR), and therapies designed to neutralize their function have shown clinical benefit [5]. In addition the androgen receptor (AR) is expressed in breast cancer in 60%–70% of tumors regardless of the ER status [6] and has been linked with a good outcome in ER positive tumors [7]. This good prognosis is associated with the impeding of the transcriptional activity mediated by the ER [7]. By contrast, in the apocrine breast cancer subtype- an ER negative tumor- the AR acts by binding to the same transcription factors as the ER does, mainly through FOXA1, leading to a luminal gene expression phenotype [8]. In this case the expression of AR is associated with worse prognosis [8]. Recently, using gene expression analyses TNBC has been classified in subtypes including one termed luminal androgen that was enriched in genes related to this pathway [9].

Testosterone and particularly dihydrotestosterone (DHT) are the main activators of the androgen receptor [10]. Upon ligand binding AR translocate to the nucleus were it acts transcriptionally [10,11]. Androgens are the major sex hormones in males being produced at different levels but mainly in sex-related tissues [11]. In addition, in females, androgens are also formed at the suprarenal gland, having a functional role after the deprivation of estrogens produced in the menopause [11].

The control of AR is mediated at different levels, but signalling pathways play a key role by stabilizing or enhancing its transcriptional activity. In prostate cancer, membrane receptor tyrosine kinases (RTK) have been shown to modulate the AR expression. For instance, overexpression of HER2 in cell lines of prostate cancer results in increase AR activity and stability [12]. Furthermore in breast cancer, a recent study has linked the activation of HER2 with the expression of AR [13]. Of note, the PI3K pathway is the key node for the signal transmission of the stimuli from membrane RTKs in relation to the control of the AR in prostate cancer [14,15]. To this regard, in breast cancer, mutations at the kinase domain of the PI3KCA gene were linked with higher expression of the AR [16].

In TNBC, expression of the epidermal growth factor receptor (EGFR) or Platelet-derived Growth Factor Receptor (PDGFRβ) has been described using immunohistochemical techniques [17]. A frequent activation of the EGFR and other receptor (Met and Eph2) as well as non-receptor (Lyn, Src family kinases) tyrosine kinases has been confirmed using a profiling of tyrosine phosphorylated proteins [18]. Moreover using shRNA library screening several kinases, including the EGFR and HER2, were identified as activated in TNBC [19].

The mechanism of action of androgens in TNBC remains controversial and the relationship between activated RTKs and the expression of AR has not been fully explored, although some recent reports have suggested the AR as a potential therapeutic target [13].

In order to contribute to a better understanding of the role of AR in TNBC, the aim of this study is to evaluate the expression of the AR in TNBC and its relationship to the activation status of RTKs and its downstream routes.

## Methods

### Reagents and antibodies

Cell culture media and supplements (fetal bovine serum (FBS), glutamine, penicillin/streptomycin) were purchased from Invitrogen (Gaithersburg, MD). The anti-pErk1/2, anti-Erk2, anti-PDGFR β, anti-pPDGFR β, anti-EGFR and anti-pEGFR antibodies were purchased from Santa Cruz Biotechnology (Santa Cruz, CA). The anti-Akt and anti-AR antibodies were from Cell Signalling Technologies (Beverly, MA). The anti-phosphorylated Akt (Serine 473) antibody was generated against the sequence RPHFPQFpS473YSAS (p = phosphorylated) [20].

### Cell culture

All cell lines were cultured at 37°C in a humidified atmosphere in the presence of 5% CO_2_–95% air. Cells were grown in DMEM or in RPMI medium containing a high glucose concentration (4,500 mg/liter) supplemented with antibiotics (penicillin at 100 U/ml, streptomycin at 100 μg/ml), glutamine 2 mM and 10% FBS. Cells were treated with different drugs: PD98059 (50 μM), BEZ235 (0.5 μM), BIC (20 μM), Imatinib (10 μM), Lapatinib (10 μM) and DHT (50nM).

### Identification of human samples and studies

Human samples were obtained from the tumor bank of the Salamanca University Hospital and Albacete University Hospital following institutional and ethical guidelines. All patients signed the study consent form. TNBC samples were defined as those with HER2 negative by inmunohistochemistry (IHC), HER2 of 0 or 1+ or a negative fluorescence in situ hybridization (FISH). HER2 amplification (evaluated by FISH DAKO HER2 FISH pharmDxTM Kit, DakoCytomation, Glostrup, Denmark A/S) was defined as a HER2-chromosome 17 ratio of < .2.0, as required by guidelines. Hormone receptor (HR) negative was defined as follow <10% positive cells by IHC for both estrogen receptor (ER) and progesterone receptor (PR) following recommendations before 2011.

### Protein extraction from human samples

Frozen human samples were inspected by haematoxylin-eosin staining for epithelial tumor content by analysis of two slices at each end of the tumor. Only samples containing 70% epithelial tumoral cells were selected for Western analyses. The tumors were minced, washed with phosphate-buffered saline (PBS), and homogenized in ice-cold lysis buffer (140 mM NaCl; 10 mM EDTA; 10% glycerol; 2% Triton X-100; 20 mM Tris pH 7.0; pepstatin, 10 mM; aprotinin, 10 mg/ml; leupeptin, 10 mg/ml; PMSF, 1 mM; beta-glycerophosphate, 25 mM; sodium fluoride, 10 mM; and sodium orthovanadate, 10 mM) with a tight-fitting Dounce homogenizer. This homogenate was centrifuged at 10,000 g for 20 minutes at 4 uC, and the supernatants were transferred to new tubes.

### Antibody arrays

Two commercial arrays were used for the studies; the human phospho-RTK array kit (R&D Systems, Abingdon, United Kingdom) and the PathScan RTK Signaling Antibody Array Kit (Cell Signaling). The Image J 1.44 software (National Institute of Health, Bethesda, MD, USA) was used for the quantification of the different RTKs in the human phospho-RTK array kit, and the Odyssey V3.0 program for the quantification of the cell signalling intermediates.

### Western blotting

Cells were washed with phosphate-buffered saline and lysed in ice-cold lysis buffer (140 mM NaCl, 10 mM ethylenediaminetetraacetic acid, 10% glycerol, 1% Nonidet P-40, 20 mM Tris, pH 7.0, 1 μM pepstatin, 1 μg/ml aprotinin, 1 μg/ml leupeptin, 1 mM phenylmethyl sulphonyl fluoride (PMSF), and 1 mM sodium orthovanadate) [21]. Lysates were centrifuged at 10,000 × g at 4°C for 10 min, and supernatants were transferred to new tubes. Samples were then boiled in electrophoresis sample buffer and placed on 6%–15% sodium dodecyl sulfate–polyacrylamide gel electrophoresis (SDS-PAGE) gels, depending on the molecular weight of the proteins to be analyzed. After electrophoresis, proteins in gels were transferred to polyvinylidene difluoride (PVDF) membranes (Millipore Corporation). Membranes were blocked in Tris-buffered saline with Tween 20 (TBST) (100 mM Tris [pH 7.5], 150 mM NaCl, 0.05% Tween 20) containing 1% of bovine serum albumin for 1 hour and then incubated with the corresponding antibody for 2–16 hours. After washing with TBST, membranes were incubated with HRP-conjugated anti-mouse or anti-rabbit secondary antibodies for 30 minutes and bands were visualized by using ECL Western Blotting Detection System (GE Healthcare, Buckinghamshire, United Kingdom).

### Cell proliferation assays

Cells were plated in 24-well plates at 15,000–20,000 cells/well and cultured overnight in DMEM or RPMI + 10% FBS. The next day medium was replaced with DMEM or RPMI containing the different drugs. Cell proliferation was analyzed days later by an MTT-based assay as described [22]. Unless otherwise indicated, the results are presented as the mean ± standard deviation (SD) of quadruplicates of a representative experiment that was repeated at least three times.

### Revere transcription (RT)-PCR

Total RNA was extracted with RNeasy Mini Kit (Quiagen) as recommended by the supplier. cDNAs were synthesized from 5 μg of total RNA by using RevertAid H Minus First Stand cDNA Synthesis Kit (Fermentas) in a total volume of 20 μl. Reverse transcription was performed at 65° for 5 min followed by 60 min at 42°. PCR reactions were performed with 2 μl cDNA in a 50 μl volume and using Taq Polymerase (Biotools) and the following primer pairs (fragments size indicated in brackets): AR (247 bp) forward 5′-CTCACCAAGCTCCTGGACTC-3′ and reverse 5′-CAGGCAGAAGACATCTGAAAG -3′, β-actin (661 bp), forward 5′-TGACGGGGTCACCCACACTGTGCCCATCTA-3′ and reverse 5′-CTAGAAGCATTTGCGGTGGACGATGGAGGG-3′. PCR cycle conditions were 1 min at 95°C, 1 min at 55°C, and 1 min at 72°C for AR and β-actin. The number of cycles was 35 and the products were resolved on a 1% agarose gel and visualized with ethidium bromide.

The design of this study is approved by the Committee of Ethics in Research of the University hospital of Albacete (Reference PI-2010/017).

## Results

### Expression of AR in human samples and cell lines

**Figure 1:**
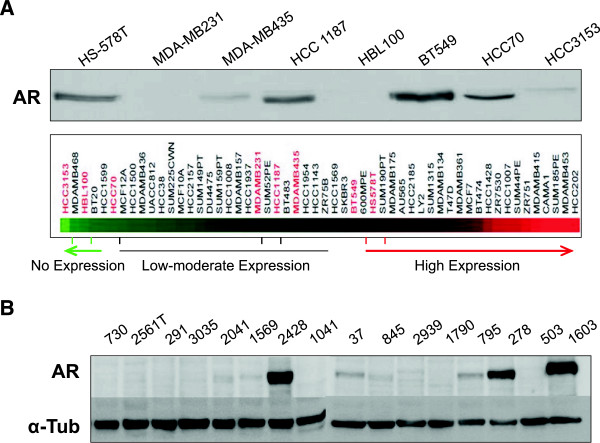
**Expression of AR in a panel of TNBC cell lines and in human samples. A)** Eight cell lines, Hs578T, MDA-MB231, MDA-MB435, HCC 1187, HBL 100, BT 549, HCC 70 and HCC 3153 were cultured and cell lysates were analyzed by western blot for AR expression. α-tubuline was used as a loading control. Expression of the AR gene from public gene expression profile data bases. **B)** Human samples of triple negative breast cancer patients were processed for protein extraction and lysates were analyzed by western blot for AR expression. α-tubuline was used as a loading control.

We used a panel of eight TNBC cell lines to evaluate the expression of the AR. Five out of eight cell lines expressed the AR, except for HBL100, HCC3153, and MDA-MB231. To corroborate these findings at a genetic level we extracted gene expression data from public libraries and evaluated the expression of the AR gene in a panel of cell lines. BT549 and HS578T (claudin-low subtype) had the highest level, HCC3153 and HCC70 (basal-like subtype) the lowest, and MDA-MB231 (claudin-low subtype) and MDA-MB435 (claudin-low subtype), had low to moderate levels (Figure 1A).

We next evaluated the expression of the AR in 16 human samples from triple negative breast cancer patients. As can be seen in Figure 1B the expression of the AR was observed in 5 out of 16 tumors (samples number: 2428, 37, 795, 278, 1603). Of note in three of them (samples number: 2428, 278 and 1603) the expression of the AR was high.

### Expression of the androgen receptor correlates by activated receptor tyrosine kinases in human samples

**Figure 2:**
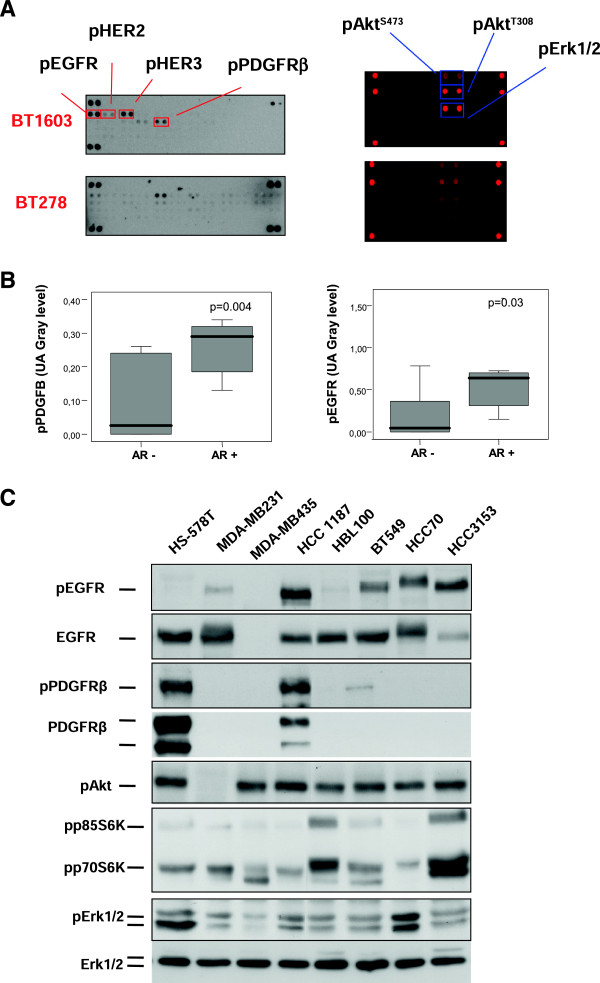
**Activation of TKRs and their downstream pathways in human samples and in TNBC cell lines. A)** Human samples of triple negative breast cancer patients were processed for protein extraction. Lysates containing 1 mg/ml protein were analyzed for the level of tyrosine phosphorylation in a panel of 42 receptor tyrosine kinases (RTKs) and citosolic kinases using antibody array kits. The levels of phosphorylated EGFR, HER2, HER3, PDGFRβ, Akt (S473), Akt (T308) and Erk1/2 are shown (indicated by arrows). **B)** Relationship between pPDGFRβ and pEGFR and AR expression in human samples. Activated PDGFRβ and EGFR obtained from the antibody array kit were measured using a gray level scale in arbitrary units. AR expression measured by western blot was classified in two groups: negative or positive. Statistical difference (p < 0.05) was analyzed using a Mann–Whitney test. **C)** Eight cell lines Hs578T, MDA-MB231, MDA-MB435, HCC 1187, HBL 100, BT 549, HCC 70 and HCC 3153 were cultured and cell lysates were analyzed by western blot for the levels of different proteins as well as their phosphorylated forms.

Given the fact that one of the mechanisms that control the expression of the AR in prostate cancer is linked with kinase signaling pathways, we evaluated if the activation of membrane RTKs or their downstream pathways could be associated with the expression of the AR. For this purpose, we analyzed the expression of 42 RTKs and downstream effectors using phospho-specific antibody arrays in the 16 human samples from TNBC patients. Two examples are shown in Figure 2A and all date available in Additional file 1: Figure S1.

Two RTKs, the PDGFRβ and EGFR, were phosphorylated in an important number of patients (more than 20%), and their activation status correlated at a significant level with the expression of the AR measured by western-blot (Figure 2A-B) (Mann–Whitney test: EGFR and AR: p = 0.03; PDGFRβ and AR: p = 0.004). We did not identify any association between the activation status of other RTKs and the expression of the AR (data not shown). Using a similar approach we studied the kinase activation profile of proteins involved in the intracellular signaling mediated by RTKs (Figure 2A, right arm). Of note pAKT (pAKTS473, pAKTT308) and pErk1/2 were expressed in more than 40% of the samples. Indeed, pAKTS473 was expressed in around 90% of the tumors. Additional file 1: Figure S1 describes the composition of both arrays and the activation profile of these kinases. No differences were observed between any of the intracellular signaling proteins- AKT or Erk1/2- and the expression of the AR (Additional file 2: Figure S2, upper panel). This lack of association could be explained by the limited sample size used and the wide expression of these activated kinases (pAKTS473 is expressed in 90% of samples and pErk1/2 in more than 40%). However, some samples with a strong activation of pAKTS473 also showed higher expression of the AR including samples BT278, BT1603 and BT2428 (Additional file 2: Figure S2, middle and lower panel).

### Activation of tyrosine kinase receptors or downstream pathways in relation to the expression of the AR in cell lines

We then studied the expression of RTKs and downstream pathways in the panel of TNBC cell lines. EGFR was activated in HCC1187, BT 549, HCC 70 and HCC 3153; and PDGFR was activated in HS578 and HCC 1187. All these cell lines described, except HCC3153, expressed the AR (Figure 2C).

In addition we studied downstream activated pathways including AKT-mTOR and Erk1/2. All cell lines had activation of pAKT except MDA-MB231. pS6 and pErk1/2 were expressed in all cell lines at different amount levels (Figure 2C). The high presence of phosphorylated AKT and Erk1/2 matched the results observed using human samples, suggesting that these cell lines could be used as a representative *in vitro* model. However, the increased existence of activated AKT and Erk1/2 in most of these cell lines made difficult to identify any association between the expression of the AR and the activation of these pathways.

### Modulation of AR expression by pharmacological inhibition

Given the association observed between some RTKs and downstream pathways with the expression of the AR in human samples and cell lines, we evaluated if the pharmacological inhibition of these receptors could modify the expression of the AR.

**Figure 3:**
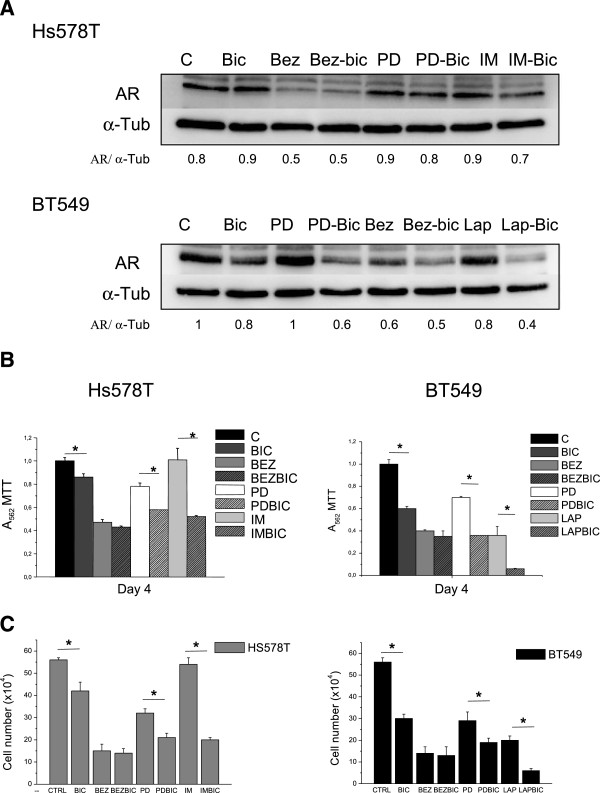
**Effect of PI3K/mTOR, Erk1/2 and EGFR/PDGFRβ inhibitors alone or in combination with bicalutamide on the AR expression and cell proliferation in Hs578T and BT549. A)** Effect of drugs on AR expression in Hs578T and BT549. Cells were cultured and treated with drugs for 24 h. Cell lysates were analyzed by western blot for AR expression. α-tubuline was used as a loading control. **B)** Effect of drugs on cell proliferation in Hs578T and BT549. MTT metabolization was performed after 4 days to evaluate cell proliferation. Control cells were untreated. Statistical difference (* = p < 0.05, Bic versus control or drug combination versus drug alone.) was analyzed using a Test T. **C)** Effect of drugs on cell proliferation in Hs578T and BT549. Cell counting was performed after 4 days to evaluate cell proliferation. Control cells were untreated. Statistical difference (* = p < 0.05, Bic versus control or drug combination versus drug alone.) was analyzed using a Test T.

For this purpose we used two cell lines; BT549, with constitutive activation of EGFR; and HS578T, with activation of PDGFRβ. Both cell lines have activation of AKT, S6 and Erk1/2, being HS578T a cell line with more activation of Erk1/2. Treatment with imatinib mesylate, a PDGFβ inhibitor, do not decreased the amount of the AR in HS578T; and a similar effect was observed for lapatinib, an EGFR inhibitor, in BT549 (Figure 3A).

As EGFR and PDGFR signal through downstream pathways, mainly the PI3k-mTOR and the Erk1/2 pathway, and these routes have also been implicated in the androgen-independent control of the AR in prostate cancer, we evaluated if the inhibition of these central nodes could have more effect on the expression of the AR than individual inhibition of RTKs. Using the same two models, we observed that the administration of PD98059, a MEK inhibitor that inhibits Erk1/2, did not reduce the amount of the AR (Figure 3A). By contrast the PI3K-mTOR inhibitor BEZ235 reduced substantially the amount of the AR in both cell lines (Figure 3A).

We next explored the action of the anti-androgen bicalutamide when combined with inhibitors of EGFR, PDGFRβ and the PI3K-mTOR and Erk1/2 pathways. Interestingly we observed that the concomitant administration of bicalutamide with EGFR, PDGFRβ and MEK inhibitors reduced the amount of the AR compared to each agent alone (Figure 3A). This finding was not observed when combining a PI3K-mTOR inhibitor with bicalutamide.

### Effects on proliferation of tyrosine kinase inhibitors alone or in combination with bicalutamide

We decided next to evaluate the effect on proliferation of pharmacological inhibitors of these receptors and pathways alone or in combination with antiandrogens. Bicalutamide alone reduced the MTT uptake in both cell lines (Figure 3B). Interestingly, in both cell lines the combination of bicalutamide with MEK inhibition was able to produce a greater anti-proliferative effect than each agent given alone (p < 0.05). Interestingly, this combination effect was not observed for BEZ235. In HS578T, imatinib was unable to reduce the MTT uptake but increased the anti-proliferative effect of bicalutamide; and in BT549 lapatinib augmented the action of bicalutamide in a similar manner (Figure 3B). A similar effect was observed counting cells (Figure 3C).

### Action of DHT on the expression of the AR

**Figure 4:**
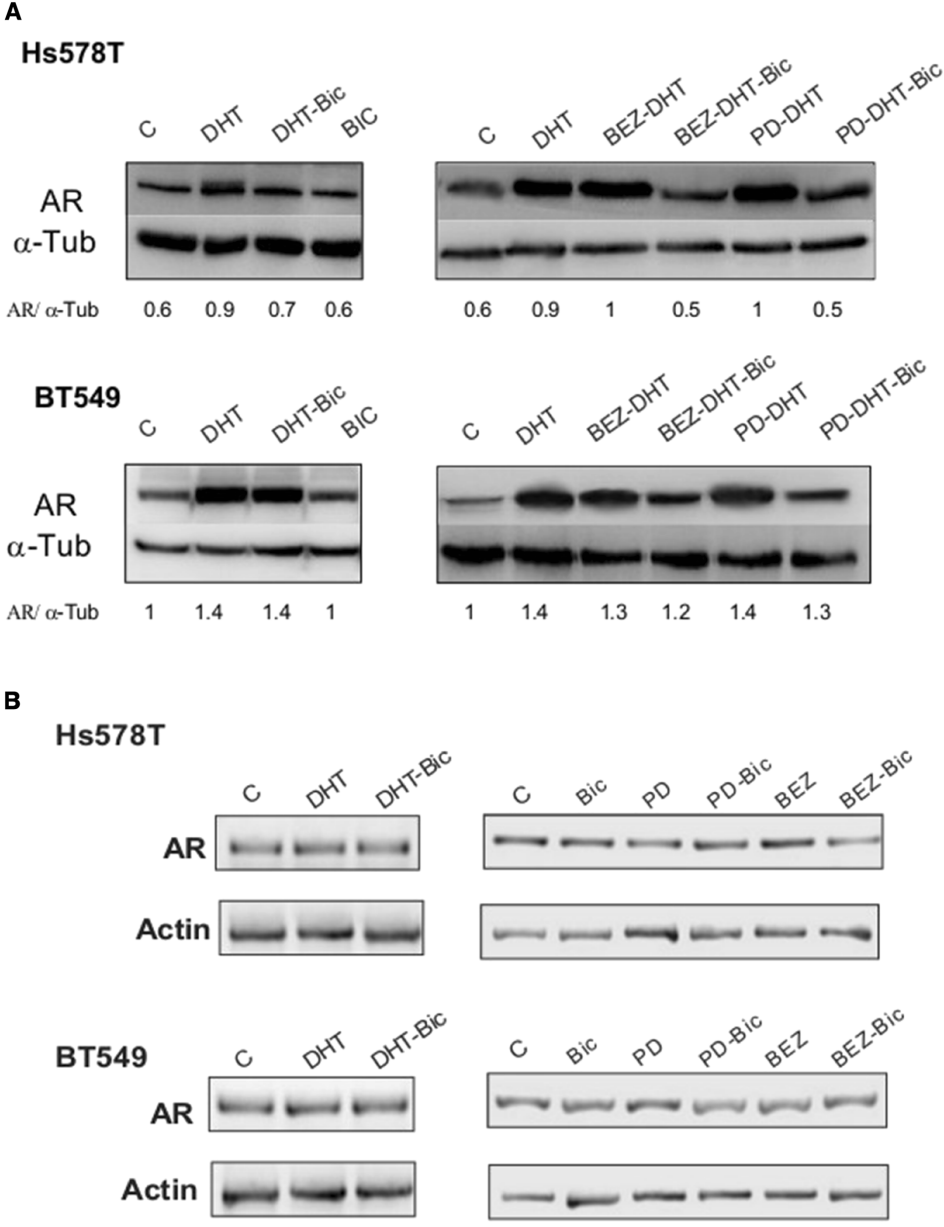
**Effect of DHT and bicalutamide alone and in combination with PI3K/mTOR, Erk1/2 and EGFR/PDGFRβ inhibitors in AR expression and cell proliferation. A)** Effect of DHT and bicalutamide, BEZ and PD in alone and combined on AR expression in Hs578T and BT549. Cells were cultured and treated with drugs for 24 h. Cell lysates were analyzed by western blot for AR expression. α-tubuline was used as loading control. **B)** In parallel, total RNA was extracted and subjected to RT-PCR. Subsequently, amplification products were resolved on agarose gel to determine AR mRNA levels. Actin was used as protein loading control and mRNA as gene control.

We then evaluated the action of DHT and bicalutamide on the expression of AR and how the inhibition of these kinases can modulate its expression. Interestingly administration of DHT increased the amount of the AR in HS578T and BT549 (Figure 4A). Administration of bicalutamide did not reduce the expression of the AR when given alone or in combination with DHT in BT549. However, in HS578T bicalutamide partially prevented the amount increase of the AR in the presence of DHT (Figure 4A). The amount of the AR gene measured by polymerase chain reaction (PCR), were not modified by the administration of DHT suggesting that this is a posttranscriptional mechanism (Figure 4B). When we evaluated the action of Erk1/2 and PI3K inhibitors on the expression of AR after stimulation with DHT, we observed that the concomitant administration of BEZ235 was not able to reduce AR expression as observed previously when given alone (Figure 3). In the presence of DHT, only bicalutamide is able to reduce the expression of AR produced by DHT (Figure 4A). Similarly, BEZ235 or PD98059 given alone; or BEZ235 and PD98059 in combination with bicalutamide did not modify the amount of the AR gene (Figure 4B).

## Discussion

In the present work, we describe the expression of the AR in TNBC and its relation to RTKs and their downstream signal routes. It is also interesting to remark that the expression of the AR was studied in cell lines not representative of a luminal androgen receptor gene expression subtype [9].

In prostate cancer evidence support the link between the activation of RTKs, like the insulin-like growth factor, keratinocyte growth factor, EGFR or HER2, and the transactivation of the AR in the absence of androgens [12,23,24]. Particularly signaling through ErbB receptors and HER2 augment the transcription of the AR; and activation of HER2 and HER3 through the HER3-ligand Neuregulin increases the amount of the AR [12,23]. Therefore inhibition of these kinases sensitizes cells to androgen withdrawal [23,25]. In estrogen negative breast cancer, the AR regulates the HER2 function suggesting that antiandrogens is a potential therapeutic approach [13]. Indeed, studies in breast cancer using human samples confirm that those tumors that are HER2 positive express higher amounts of the AR [26].

In our study we show a correlation between activated membrane tyrosine kinase receptors and the expression of the AR in TNBC. It is known that in triple negative tumors EGFR and PDGFRβ are phosphorylated and present in a significant number of tumors [19,27]. If extrapolating from mechanisms observed in prostate cancer, is not surprising to identify a correlation between activated RTKs and the expression of the AR. Interestingly this association was also observed in a panel of cell lines, and treatment with specific tyrosine kinase inhibitors against them reduced slightly the amount of the AR, particularly with lapatinib in BT549 when combined with bicalutamide. A slightly similar effect was observed for imatinib and bicalutamide in HS578T. As the PI3K/AKT pathway and the MAPK pathway are key intracellular signaling nodes from RTKs, we decided to evaluate their role in the activation of the AR. It is known that targeting key nodes has a more profound effect on the inhibition of deregulated functions that the inhibition of single receptors [28]. In prostate cancer the PI3K/AKT pathway has been described as a key regulator in the transcription of the AR [12], and in breast cancer mutations at the PIK3CA gene were associated with expression of the AR in patients [16]. Our results show that the inhibition of the PI3K/mTOR pathway with BEZ235 decreased the amount of the AR in the absence of androgens.

It was interesting to find how the concomitant administration of an Erk1/2 inhibitor with bicalutamide reduced the amount of the AR in a more profound manner than when each agent was given alone. This effect was also observed when using inhibitors of EGFR and PDGFRβ.

We then studied the effect on proliferation of these combinations. Although an important effect was noted with BEZ235 given alone, no effect on proliferation was found when associated with an anti-androgen. Furthermore, the concomitant administration of PD98059 plus bicalutamide produced a profound arrest in proliferation compared to each agent given alone. This effect suggests that the inhibition of the MAPK pathway facilitates the anti-proliferative effect of anti-androgens. A synergistic interaction between anti-androgens and Erk1/2 inhibitors has been described in other models including apocrine breast cancer, but not in TNBC [29]. A similar result was observed for the combination of lapatinib and bicalutamide in BT549 and for imatinib in HS578T.

Stimulation with DHT increased the amount of the AR, and this was not prevented by the administration of either an inhibitor of PI3K or Erk1/2; showing that the mechanism associated with the expression of the AR by androgens is independent of a kinase control. Furthermore, effects on the expression of AR after stimulation with DHT or when administering kinase inhibitors were independent of the expression of the AR gene, suggesting a posttranscriptional mechanism.

Our findings have some potential therapeutic translations. It has been suggested that the AR is a potential druggable target in TNBC and indeed, clinical studies are currently evaluating the role of androgen deprivation in the treatment of patients with breast cancer [30]. In our work we identify a relationship between the expression of the AR and the activation of membrane kinases like EGFR and PDGFRβ. Furthermore, the concomitant inhibition EGFR and PDGFRβ with bicalutamide increased the antiproliferative effect compared with each agent given alone. A similar effect was observed for the administration of the Erk1/2 inhibitor with bicalutamide.

## Conclusions

We observed a positive correlation between the activation of EGFR and PDGFRβ and the expression of the AR. Inhibition of PI3K pathway decreased the total amount of the AR. Inhibition of Erk1/2, PDGFRβ and EGFR decreased the expression of the AR when given concomitantly with bicalutamide and had an additive anti-proliferative effect, suggesting that administration of anti-androgens in TNBC should be given concomitantly with inhibitors of these kinases. Modification of AR expression by these kinase inhibitors was posttranscriptionally regulated. In conclusion, our results describe mechanisms associated with the control of the AR expression in TNBC, and demonstrate a potential therapeutic opportunity for the combination of anti-androgens with RTK inhibitors and its downstream routes.

## Competing interests

The authors declare that they have no competing interests.

## Authors’ contributions

AO carried out the design and drafting the manuscript. AP participates in the design of the manuscript. MDC-L carried out the molecular studies, participated in the design and drafting manuscript. JC Montero, JC Morales and AP participate in the molecular studies. All authors read and approved the final manuscript.

## Pre-publication history

The pre-publication history for this paper can be accessed here:

http://www.biomedcentral.com/1471-2407/14/302/prepub

## Supplementary Material

Additional file 1: Figure S1 Human Phospho Kinase Array kit including Receptor Tysosine Kinases and downstream proteins in human samples from triple negative breast cancer patients. Lower pannels describe the exact possition of each protein in duplicate. For more details see the Methods section. Click here for file

Additional file 2: Figure S2 Relationship between pAKTS473, pAKTS308, pErk1/2 and AR expression in human samples using data from the kinase arrays. AR expression was measured by western blot (as described in Figure 1) and classified in two groups: negative or positive. Lower paneel shows three representative examples of human patients where pAKT and pErk1/2 was activated. Click here for file
