# Supplementary material for: Phospho-kinase profile of triple negative breast cancer and androgen receptor signaling
Source: BMC Cancer. 2014 Apr 30;14:302. doi: 10.1186/1471-2407-14-302 (PMC4021223; doi:10.1186/1471-2407-14-302)
Supplement: Additional file 1: Figure S1 — Human Phospho Kinase Array kit including Receptor Tysosine Kinases and downstream proteins in human samples from triple negative breast cancer patients. Lower pannels describe the exact possition of each protein in duplicate. For more details see the Methods section. [file 1471-2407-14-302-S1.pdf]

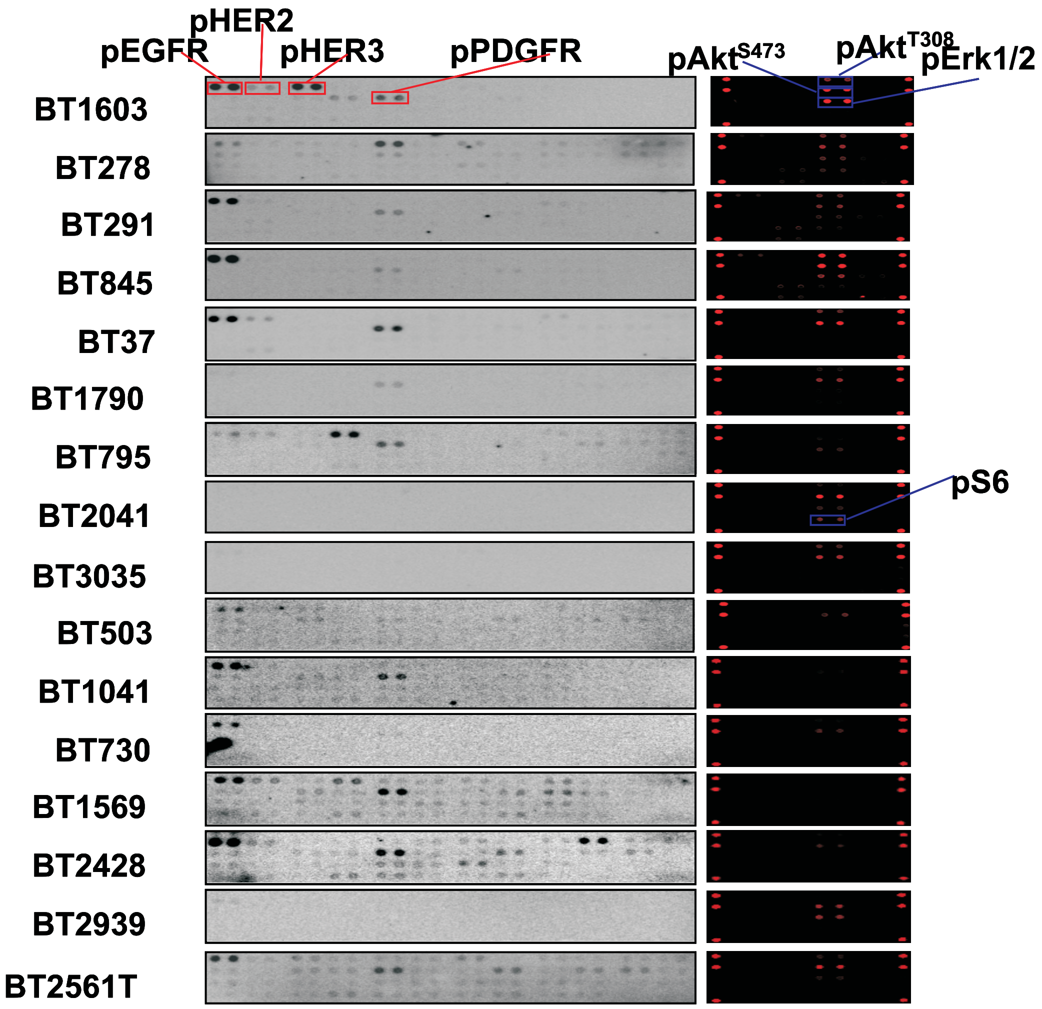

Coordinate	RTK	Coordinate	RTK
A1,A2	EGFR	C1,C2	Tie-2
A3,A4	Erbb2	C3,C4	TrkA
A5,A6	ErbB3	C5,C6	Trkb
A7,A8	ErbB4	C7,C8	Trkc
A9,A10	FGFR1	C9,C10	VGEFR1
A11,A12	FGFR2a	C11,C12	VGEFR2
A13,A14	FGFR3	C13,C14	VGEFR3
A15,A16	FGFR4	C15,C16	MuSK
A17,A18	Insulin R	C17,C18	EphA1
A19,A20	IGF-1R	C19,C20	EphA2
A21,A22	Axl	C21,C22	EphA3
A23,A24	Dtk	C23,C24	EphA4
B1,B2	Mer	D1,D2	EphA6
B3,B4	HGFR	D3,D4	EphA7
B5,B6	MSPR	D5,D6	EphB1
B7,B8	PDGFRa	D7,D8	EphB2
B9,B10	PDGFRb	D9,D10	EphB4
B11,B12	SCFR	D11,D12	EphB6
B13,B14	Flt-3	D13,D14	M IgG1
B15,B16	M-CFSR	D15,D16	M IgG1 2A
B17,B18	c-Ret	D17,D18	M IgG1 2B
B19,B20	ROR1	D19,D20	G IgG
B21,B22	ROR2	D21,D22	PBS
B23,B24	Tie-1		

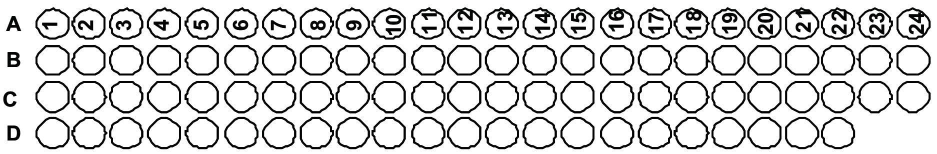

Coordinate	Substrate	Coordinate	Substrate
A1	Control +	D1	Control -
A2,A3	EphA1	D2,D3	EphB1
A4,A5	EphB4	D4,D5	Tie2/TEK
A6,A7	Akt T308	D6,D7	S6 S235/236
A8,A9	IRS-1	D8,D9	Lck
A10	Control +	D10	Stat3
B1	Control +	E1	Control +
B2,B3	EphA2	E2,E3	EphB3
B4,B5	Tyro-3/Dtk	E4,E5	VEGFR2
B6,B7	Akt S473	E6,E7	C-Abl
B8,B9	Zap-70	E8,E9	Stat1
B10	Control +	E10	Control +
C1	Control -		
C2,C3	EphA3		
C4,C5	Axl		
C6,C7	ERK1/2		
C8,C9	Src		
C10	Stat3		

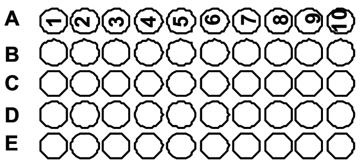

Supplementary Figure 1
