# Supplementary figures and images for: Phospho-kinase profile of triple negative breast cancer and androgen receptor signaling
Source: BMC Cancer. 2014 Apr 30;14:302. doi: 10.1186/1471-2407-14-302 (PMC4021223; doi:10.1186/1471-2407-14-302)

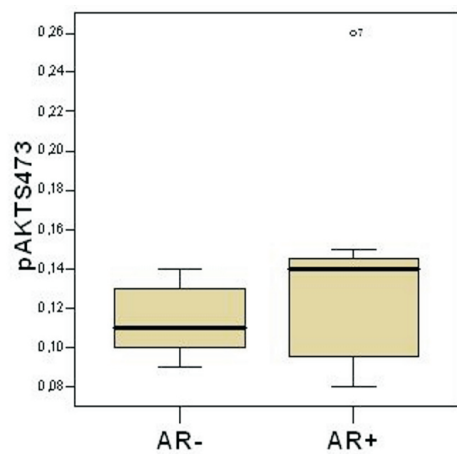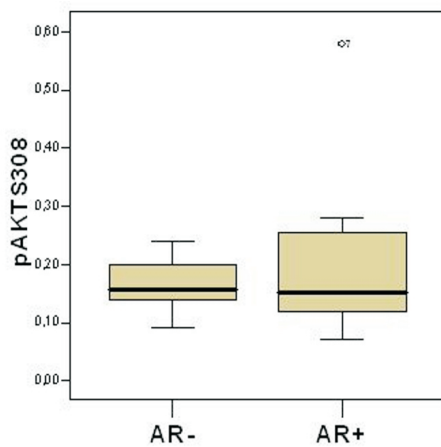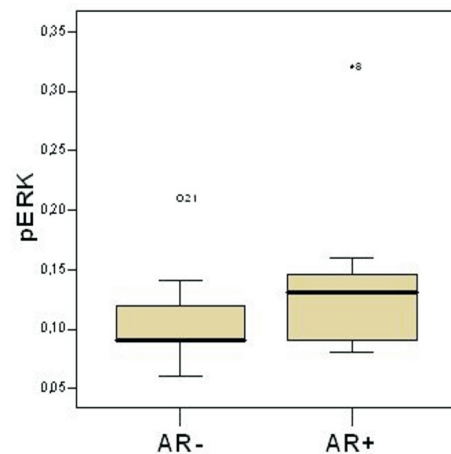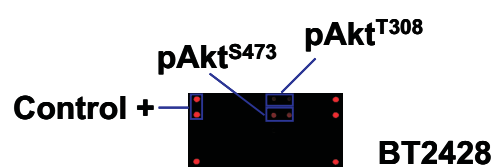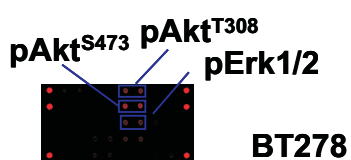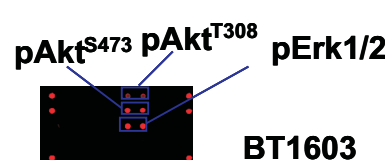

Supplementary Figure 2

Supplement: Additional file 2: Figure S2 — Relationship between pAKTS473, pAKTS308, pErk1/2 and AR expression in human samples using data from the kinase arrays. AR expression was measured by western blot (as described in Figure 1) and classified in two groups: negative or positive. Lower paneel shows three representative examples of human patients where pAKT and pErk1/2 was activated. [file 1471-2407-14-302-S2.pdf]
